# The expression and biological function of chemokine CXCL12 and receptor CXCR4/CXCR7 in placenta accreta spectrum disorders

**DOI:** 10.1111/jcmm.14990

**Published:** 2020-01-28

**Authors:** Yu Long, Yonghua Jiang, Jingjing Zeng, Yiwu Dang, Yue Chen, Jueying Lin, Hongwei Wei, Hongwei Xia, Junqing Long, Cuizhen Luo, Zhiwei Chen, Yaling Huang, MuJun Li

**Affiliations:** ^1^ Department of Gynecology and Obstetrics The First Affiliated Hospital of Guangxi Medical University Nanning China; ^2^ Center for Genomic and Personalized Medicine Guangxi Medical University Nanning China; ^3^ Department of Pathology The First Affiliated Hospital of Guangxi Medical University Nanning China; ^4^ Department of Gynecology and Obstetrics The First People’s Hospital of Nanning Nanning China; ^5^ Department of Gynecology and Obstetrics The Maternal & Child Health Hospital the Obstetrics & Gynecology Hospital of Guangxi Zhuang Autonomous Region Nanning China; ^6^ School of Clinical Medicine Guangxi Medical University Nanning China; ^7^ Wuming District Center for Disease Prevention and Control Nanning China; ^8^ Department of Reproductive Center The First Affiliated Hospital of Guangxi Medical University Nanning China

**Keywords:** CXCL12, CXCR4/CXCR7, Function, Invasion, placenta accreta spectrum disorders, Trophoblast

## Abstract

**Objectives:**

Investigation of mechanism related to excessive invasion of trophoblast cells in placenta accreta spectrum disorders (PAS) provides more strategies and ideas for clinical diagnosis and treatment.

**Materials and Methods:**

Blood and placental samples were collected from included patients. The distribution and expression of CXCL12, CXCR4 and CXCR7 proteins in the paraffin of placental tissue in the included cases were analysed, and we analyse the downstream pathways or key proteins involved in cell invasion.

**Results:**

Firstly, our results determined that CXCL12 and CXCR4/CXCR7 were increased in extravillous trophoblastic cell (CXCL12: *P* < .001; CXCR4: *P* < .001; CXCR7: *P* < .001), and the expression levels were closely related to the invasion depth of trophoblastic cells. Secondly, CXCL12 has the potential to become a biochemical indicator of PAS since the high expression of placental trophoblast CXCL12 may be an important source of blood CXCL12. Using lentivirus‐mediated RNA interference and overexpression assay, it was found that both chemokine CXCL12 and receptor CXCR4/CXCR7 are associated with regulation of trophoblast cell proliferation, migration and invasion. Further results proved that through the activating the phosphorylation and increasing the expression of MLC and AKT proteins in the Rho/rock, PI3K/AKT signalling pathway, CXCL12, CXCR4 and CXCR7 could up‐regulate the expression of RhoA, Rac1 and Cdc42 proteins to promote the migration and invasion of extravillous trophoblastic cell and ultimately formate the placenta accrete compare to the normal placenta.

**Conclusions:**

Our research proved that trophoblasts may contribute to a PAS‐associated increase in CXCL12 levels in maternal blood. CXCL12 is not only associated with biological roles of PAS, but may also be potential for prediction of PAS.

## 1 INTRODUCTION

Placenta accreta spectrum disorders (PAS) is characterized by excessive invasion of the chorionic villi in the myometrium, resulting in severe haemorrhage during and after delivery. PAS is one of the main causes of perinatal emergency hysterectomy and maternal death. At present, studies on the pathogenesis of PAS mainly focus on the myometrial scar,^1^ the loss or abnormal function of decidua^2^ and the abnormal angiogenesis at the invasive site^3^, ^4^ but these could only partially explain PAS. The specific molecular mechanism underlying over‐invasion phenomenon of PAS remains unclear.

Chemokine is a kind of small molecule protein with chemotaxis among members of cytokine superfamily, the molecular weight of which is approximately 8‐10 kD, and it promotes the cell migration.^5^ At present, more than 50 kinds of low molecular weight chemokines have been found. According to the number and spatial sequence of the N‐terminal semi‐desinine residues, chemokines can be categorized for four families: CXC, CC, CX3C and C family.^6^ The expression of chemokine proteins is selectively induced on the surface of target cells through binding to chemokine receptors.^7^ Basically, a chemokine may bind to multiple receptors, and a chemokine receptor can also recognize multiple chemokines, which constitutes a complex network and widely participates in cellular immunity, growth and development, inflammation and other physiological functions.^8^

The consequence of CXCL12 with its receptor CXCR4 or CXCR7 has become one of the hotspots in the study of the development of various malignant tumours.^9^ One of the important roles of CXCL12‐CXCR4/CXCR7 is to regulate the adhesion, metastasis, colonization, angiogenesis and proliferation of tumour cells to endothelial cells.^10^ Given the biological behaviour of trophoblasts similar to cancer cells, it has been indicated that CXCL12 and its receptors also mediated trophoblasts differentiation, invasion, and proliferation. In terms of the maternal‐foetal interface of early pregnancy, CXCL12 and its receptors have attracted much attention to regulate the balance between trophoblast cells and decidual cells.^11^ CXCL12‐mediated regulation is associated with trophoblast cells proliferation, differentiation, invasion and uterine spiral arterial remodelling in early pregnancy.^12^ Abnormal regulation of CXCL12 and its receptors gives rise to chemical placental diseases (IPD), such as abortion, foetal growth and development restriction, and preeclampsia.^13^ The function of CXCL12 with its receptors varies in different stages of pregnancy and different diseases, and the functional role of CXCL12‐CXCR4/CXCR7 in PAS remains to be determined. Our study therefore preformed clinical research on PAS of trophoblast cells excessive invasion and function analysis of EVT cells in vitro, seeking to provide theoretical basis and new direction for the diagnosis and treatment of PAS.

## 2 MATERIALS AND METHODS

### 2.1 Patients

The study was approved by the ethics committee of the first affiliated hospital of Guangxi Medical University. All the pregnant women enrolled in this study had written informed consents after a detailed explanation. From January 2016 to September 2017, pregnant women diagnosed with PAS and uterine repregnancy in the first affiliated hospital of Guangxi Medical University, the first people's hospital of Nanning, Guangxi maternal and child health hospital and Guangxi maternity hospital were selected for the study. A total of 33 PAS cases (gestational age: 36.45 ± 1.09 weeks) were collected, including 9 cases of placental accreta, 14 cases of placental increta and 10 cases of placental percreta. Thirty‐three healthy pregnant women with placenta (gestational age: 36.52 ± 1.09 weeks) due to caesarean section scar were selected as the normal control group. Blood and placental samples were collected from both groups.

The inclusion conditions of PAS group: (a) PAS with pathological diagnosis; (b) The pregnancy was longer than 28 weeks. The criteria of depth of PAS: (a) Pathological examination of total hysterectomy; (b) In the case of uterine preservation, the abnormal placental villi that invaded the muscular layer were cleared by hand curettage and local excision. Pathological examination showed that the lesion site contained villi and the muscular layer of the uterus; (c) combined with surgical exploration. The pathological diagnosis of PAS was defined as follows: (a) placental accreta: the villi were directly attached to the myometrium, and there was a lack of decidua between the villi and the myometrium; (b) Placenta increta: the villi invaded the myometrium; (c) placenta percreta: villi reached the serous membrane of the uterus or to the bladder, rectum and pelvic other organs and tissues through the myometrium. Subjects with multifetal gestations, gestational hypertension, gestational diabetes mellitus, placental abruption, chorioamnionitis and medical diseases were excluded. Clinicopathological data were collected, including age, gestational age, number of pregnancies, number of births, previous labour history, previous surgical history, vaginal bleeding during pregnancy, birth weight of newborn and surgical conditions.

### 2.2 Collection and preservation of clinical blood samples and placental samples

Before pregnant women received hormone therapy or blood transfusion, 4‐6 mL venous EDTA‐blood was collected and centrifuged (110 xg) at 4°C for 15 min. According to the instruction of CXCL12 kit, the blood was centrifuged (11 000 xg) for another 10 min at 2‐8°C to completely clear away the platelets in the blood plasma. The supernatant was collected and stored at −80°C for storage. Placental samples, including uterus, partial excision or excised implanted lesion, were collected and fixed with 10% formalin immediately, for further dehydration by gradient ethanol, paraffin embedding.

### 2.3 Cell culture

The extravillous trophoblastic cell line HTR8/SVneo (presented with Dr Zhifu zhizhi from guangxi medical university.) were purchased from Nanjing Key Gen Biotech Co., Ltd. (Nanjing, China), and cultured in Dulbecco's modified Eagle's medium (DMEM) (Thermo Fisher Scientific, Waltham, MA, USA) with 10% foetal bovine serum (FBS) (GE Healthcare, Logan, UT, USA) at 37°C with 5% CO_2_.

### 2.4 RNA extraction and Quantitative real‐time PCR (qRT‐PCR)

Total RNA was extracted by using TRIzol‐Reagent (Invitrogen). cDNA was transcribed from mRNA by High‐Capacity cDNA Reverse Transcription kits (Applied Biosystems, Foster City, CA). qPCR was conducted by using ABI PRISM 7700 System and FastStart Universal SYBR Green Master kit (Roche). β‐actin was used as an internal reference gene. All primers used in this study was purchased from Sangon Biotech (Shanghai, China). The relative expression level was calculated using the 2^‐ΔΔCt^ method. Primers were listed in Table 1. Experiments were carried out in triplicates.

**Table 1:** Primers used in this study

ID	Sequence (5’‐3’)
CXCL12 F	CTCCGCTGTCACCTTCCC
CXCL12 R	TGTGCCCTTCAGATTGTAGCC
CXCR4 F	GCAGCAGGTAGCAAAGTGAC
CXCR4 R	TGAAGTGTATATACTGATCCC
CXCR7 F	ATTTACAAAGCGCCGAGAGC
CXCR7 R	TGTGGGTTACAAAGCTGCCA
β‐actin F	TCTGAGGCGGGCAATCAAAT
β‐actin R	CTCCATCCTGGCCTCGCTGT

### 2.5 Immunohistochemical staining of placental tissue

The sections were incubated at 65°C about 2 hour, and dewaxed in xylene I, II for 10 minutes. After being treated with gradient concentrations of 100%, 95%, 85% and 70% ethanol for 3 minutes, the sections were immersed in boiled 0.01M citrate buffer (pH = 6.0) for 2 minutes, cooled for 30 minutes, and then washed with PBS. The sections were incubated in 3% H_2_O_2_ for 20 minutes to block the activity of endogenous peroxidase and were then washed with PBS for 3 times. The sections were blocked with normal goat serum and then incubated with primary antibodies at 4°C overnight (CXCL12, 1:100, CXCR4, 1:100, CXCR7, 1:100), then washed with PBS for 3 times. MaxVision^TM^ detection reagent was added for rapid immunohistochemistry and incubated for 20 minutes. PBS was used for washing and removed. Fresh DAB colour rendering solution (prepared according to the instructions) was added for observation under the microscope.

### 2.6 The expressions of plasma CXCL12, CXCR4 and CXCR7 via enzyme‐linked immunosorbent assay (ELISA)

The standard solution was prepared to make the standard curve, and the standard curve was drawn using CurveExpert 1.4 software for sample quantification according to the introduction of the kit (Invitrogen, Carlsbad, CA, USA). In brief, the 96‐well plate coated with the corresponding antibodies was taken, added with the standard solution or diluted plasma samples, and affixed with the sealing membrane, followed by incubation at 37°C for 90 minutes. After the liquid in the plate was discarded and dried, the biotin‐labelled antibody [anti‐CXCL12, CXCR4 or CXCR7 antibody] was added, and the plate was sealed with the sealing membrane, followed by incubation at 37°C for 60 minutes. After the plate was washed with washing solution for 4 times (3 minutes per time), ABC working solution was added, and the plate was sealed, followed by incubation at 37°C for 30 minutes. After the plate was washed again with washing solution for 4 times, tetramethyl benzidine developing solution was added, and the plate was sealed, followed by incubation at 37°C for 20 minutes at dark. Then, TMB stop buffer was added and mixed evenly. The absorbance value at 450 nm was detected using a microplate reader and substituted into the standard curve to calculate the concentrations of plasma CXCL12, CXCR4 and CXCR7 in each group.

### 2.7 Extraction of total protein from cells and Western blot

Cells in logarithmic growth period (about 80% to 90%) were taken, supernatant was discarded, and sediment was resuspended with precooled PBS for washing. RIPA cell lysate with 1 mol/L PMSF was added. About 200 μL lysate (including protease and phosphatase inhibitor) was added into each well of the 6‐well plate at a ratio of 1 mol/L, being treated in ice bath for 20 minutes. Total protein was extracted at 4°C 12 000 g for 15 minutes and treated in 100°C the boiling water for 10 minutes for subsequent Western blotting. Protein samples were separated by SDS‐PAGE and transferred to the PVDF membrane for 30 minutes. The PVDF membrane was pretreated with the blocking buffer (TBS, 0.1% tween‐20, 5% skimmed milk powder) for 1 hour at room temperature. After being washed with PBST for 3 times, the membrane was incubated with anti‐CXCL12 antibody (1:100, Abcam), anti‐CXCR4 antibody (1:100, Abcam), anti‐CXCR7 antibody (1:100, Abcam), anti‐SDF1 (1:100, Abcam), anti‐Rac1 (1:100, Abcam), anti‐CDC42 (1:100, Abcam), anti‐RhoA (1:100, Abcam), anti‐ROCK1 (1:100, Abcam), anti‐ROCK2 (1:100, Abcam) and anti‐GAPDH (1:200, Abcam) 4°C for the night, respectively. The membranes were then incubated with HRP‐labelled secondary antibody (1:5000, Kangwei century biotechnology co., Ltd) for 1 hour at room temperature after being washed with PBST. After further wash with PBST for three times, chemiluminescence detection reagent was used to develop and fix the signal. GAPDH antibody was used for normalization. The results were imaged by the gel imaging analysis system. Image J software was used to analyse the grey value of the target bands.

### 2.8 Cell transfection

OE‐CXCL12 (EX‐A0590‐Lv201), OE‐CXCR4 (EX‐Z3039‐Lv206), OE‐CXCR7 (EX‐T7363‐Lv206), shCXCL12 (HSH016669‐LVRU6GP), shCXCR4 (HSH018802‐LVRU6MP), shCXCR7 (HSH015336‐LVRU6MP), sh‐control (EX‐NEG‐Lv201) and OE‐control (EX‐NEG‐M98) were purchased from Genecopoeia company (USA). HTR‐8/SVneo cells were cultured in 6‐well plate at a density of 2 × 10^5^/well. The thawed virus solution was mixed with the complete culture medium, and 10 µg Polybrene (final concentration 5 µg/mL) was further added. The culture medium in the plate was discard, and the diluted virus mixture was added for 24 hours incubation. The complete medium was added and cultured overnight. After 72‐96 hours, fluorescence was observed under a fluorescence microscope to determine the efficiency of virus infection. After 96 hours, purinomycin was added for detection and cells were collected for RNA, protein extraction and follow‐up experiments.

### 2.9 Colony formation assay

Colony formation assay was used to evaluate the cell formation ability. 2 × 10^3^ cells were plated into each well of a 6‐well plate and incubated for 10 to 14 days. Then, the plate was gently washed and stained with crystal violet. The cell colonies were observed and counted under an inverted microscope.

### 2.10 Cell proliferation experiment

The cells were divided into five groups: Blank control group (HTR‐8/SVneo cell line), overexpressed negative control group (HTR‐8/SVneo cell line), silence negative control group (HTR‐8/SVneo cell line), overexpressed group (HTR‐8/SVneo/OE‐CXCL12, HTR‐8/SVneo/OE‐CXCR4 and HTR‐8/SVneo/ OE‐CXCR7), silence group (HTR‐8/SVneo/shCXCL12, HTR‐8/SVneo/shCXCR4 and HTR‐8/SVneo/shCXCR7 cell lines). Time points of 0, 24, 48, 72, 96 hours were included. Cell proliferation assays were performed through using Cell Counting Kit‐8 (CCK‐8) (Beyotime, Shanghai, China) in accordance with the manufacturer's protocol. Then, we measured the absorbance for each well at a wavelength of OD_450_ using an auto‐microplate reader. Experiments were repeated three times.

### 2.11 Wound healing assay

For scratch wound healing assay, cells at concentration of 2 × 10^5^ cells/mL were cultured in serum‐free medium for 24 hours, at 37℃ with 5% CO_2_ and wounded with pipette tips. Fresh medium was replaced. The wound closing procedure was observed for 48 hours. Experiments were repeated three times. The mean distance between cells was calculated with Image J software.

### 2.12 Transwell assay

The cells of each group were cultured to 60%‐80% confluency and were trypsinized. 2 × 10^5^ cells in 100 μL of serum‐free DMEM were plated into the upper chamber. DMEM (600 μL) supplemented with 20% FBS was added to the lower chamber. After being cultured for 22 hours, cells that had invaded the lower chamber were fixed with methanol and stained with 0.1% crystal violet. The number of invaded cells was observed by using an inverted microscope (magnification × 100).

### 2.13 Cell apoptosis assay

The cells of each group were cultured to 60%‐80% confluency and were digested with 0.25% trypsin. About 500 μL binding Buffer was added to suspend cells, followed by 1μL Annexin V‐PE for mixing. The Aimexin V‐PE was detected by flow cytometry (FACScan®; BD Biosciences), at the wavelength of 488 nm and emission wavelength of 578 nm. Experiments were repeated three times.

### 2.14 Statistical analysis

Statistical Product and Service Solutions (SPSS) 19.0 software (SPSS Inc, Chicago, IL, USA) was used for data processing. Data in this study were presented as mean ± standard deviation. *t* Test was used for the intergroup comparison, and chi‐square test was used for enumeration data. Continuous data from multiple groups were analysed by using one‐way ANOVA, with the Tukey's post hoc test. *P*‐values < .05 were considered statistically significant.

## 3 RESULTS

### 3.1 Patient characteristics

The comparison between PAS and normal control groups (each group n = 33) was performed. There were no significant differences in age (*P* = .957), gestational age (*P* = .160), birthweight (*P* = .626), gravidity (*P* = .211), parity (*P* = .769), and the number of prior caesarean sections (*P* = .378), but the proportion of vaginal bleeding (*P* = .021) and caesarean hysterectomy (*P* = .053) in the case group were higher than that in the control (Table 2).

**Table 2:** Clinical characteristics of the study population

Characteristics	Case (n = 33)	Control (n = 33)	*t*/χ^2^	*P* value
Age, y	32.52 ± 4.97	32.45 ± 4.45	0.054	.957
Gestational age at delivery, weeks	36.45 ± 1.09	36.52 ± 1.09	1.437	.16
Gravidity				
≤3	17 (43.59%)	22 (56.41%)	1.567	.211
>3	16 (59.23%)	11 (40.74%)		
Parity				
≤2	25 (49.02%)	26 (50.98%)	0.086	.769
>2	8 (53.33%)	7 (46.67%)		
Number of prior Caesarean sections				
0 (no prior Caesareans)	3 (9.09%)	0 (0.00%)		.378
1 prior Caesarean	25 (75.76%)	27 (81.82%)		
2 prior Caesareans	5 (15.15%)	6 (18.18%)		
Vaginal bleeding (≥1 episode)	9 (27.27%)	2 (6.06%)	5.345	.021
Birthweight, g	2875.45 ± 310.60	2928.09 ± 447.26	0.641	.626
Caesarean hysterectomy	5 (15.15%)	0 (0.00%)		.053

### 3.2 The expression of CXCL12, CXCR4 and CXCR7 in PAS and normal pregnant placental tissues

**Figure 1:**
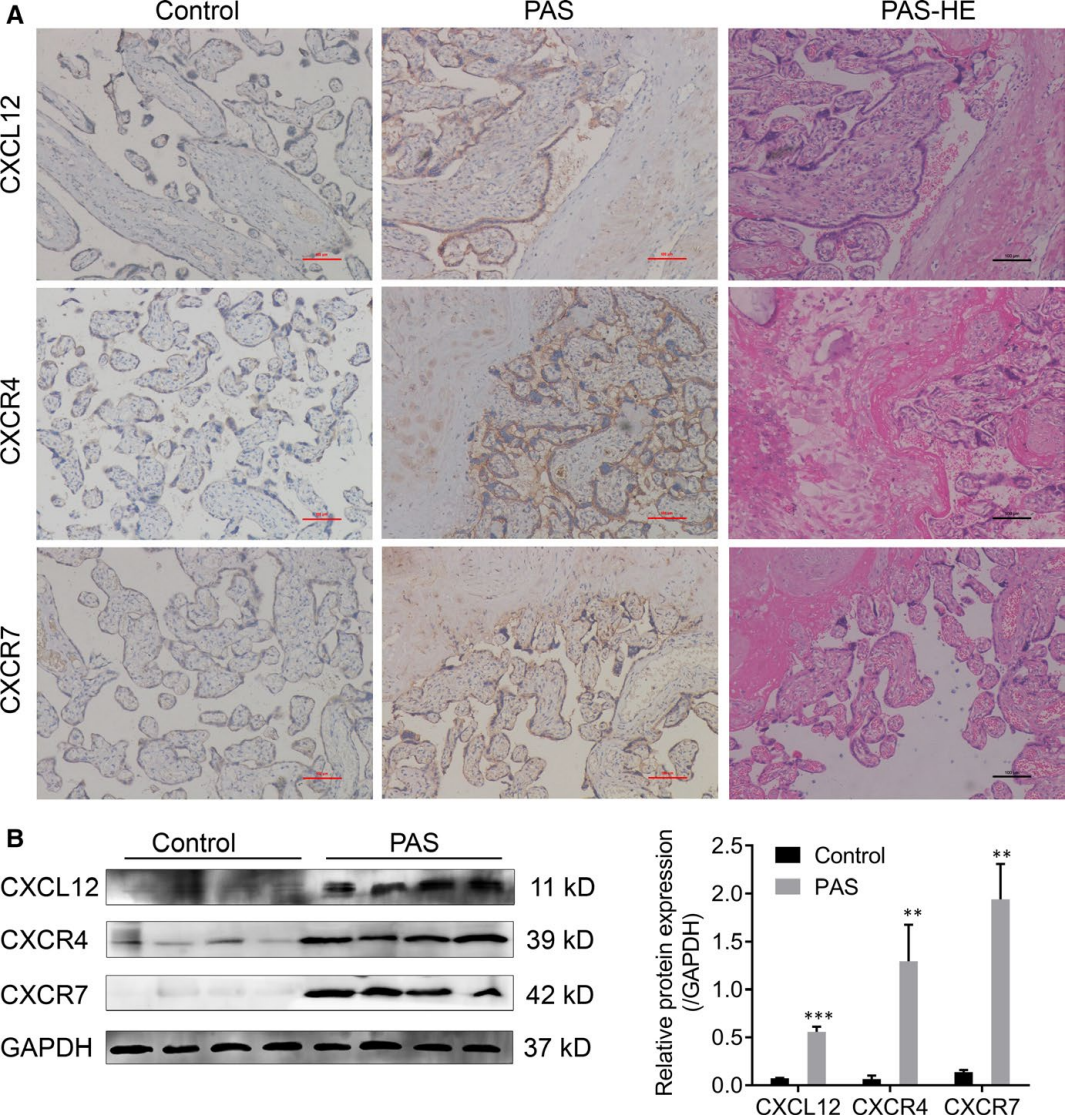
The expression of CXCL12, CXCR4 and CXCR7 in PAS and normal pregnant placental tissues. Immunohistochemical staining (A) and Western blot (B) showed the up‐regulation of CXCL12, CXCR4 and CXCR7 in PAS and normal pregnant placental tissues (Control)

Immunohistochemical staining was used to compare the expressions of CXCL12, CXCR4/CXCR7 in the internal or external trophoblast cells and placenta implant sites that were determined during surgery and histopathological section (Figure 1, Table 3). Yellow colour appeared in membrane or cytoplasm was considered as positive cells. The percentages of positive cells at 0%, 1%‐25%, 26%‐50%, 51%‐75% and >75%, respectively, were scored as 0 (zero), 1 (weak), 2 (middle), 3 (strong) and 4 for evaluation (Figure S1). Positive and negative controls were set during staining. Human colon cancer tissue, spleen tissue and lymph node tissue were used as CXCL12 CXCR4 and CXCR7 positive control, respectively, the Goat‐isotype and rabbit isotype antibody were used as negative control for placenta tissues (Figure S2). Results indicated that the levels of CXCL12, CXCR4 and CXCR7 at the implant site were higher than those of the control group (CXCL12: *P* < .001; CXCR4: *P* < .001; CXCR7: *P* < .001). Moreover, Western blot analysis also confirmed the up‐regulation of CXCL12, CXCR4 and CXCR7 in PAS. It was further subdivided into the types of placental accreta, placental increta and placental percreta, and immunohistochemical staining was utilized to detect the expressional differences of CXCL12 and CXCR4/CXCR7 proteins in trophoblast cells of all types (Table 4). Results revealed that there were significant differences in CXCL12, CXCR4 and CXCR7 among the three subgroups of percreta, increta and accreta. The pairwise comparison results showed that the expressions of CXCR4 and CXCR7 of pregnant women in the percreta group were higher than those in the increta group (CXCR4: *P* = .047; CXCR7: *P* = .023), and CXCL12, CXCR4 and CXCR7 scores of pregnant women in the percreta group were significantly higher than those in the accreta group (CXCL12: *P* = .007; CXCR4: *P* = .001; CXCR7: *P* = .005).

**Table 3:** Staining intensity scores by histologic diagnosis

	Case (n = 33)	Control (n = 33)	*Z*	*P* value
CXCL12	8.42 [4,12]	3.70 [2,8]	4.646	<.001
CXCR4	9.07 [3,12]	3.177 [2,6]	4.945	<.001
CXCR7	7.67 [3,12]	2.96 [2,8]	4.889	<.001

Note

The values in brackets indicated the minimum and maximum values of the immunohistochemical scores, respectively.

**Table 4:** Staining intensity scores by depth of invasion

	Percreta	Increta	Accreta	*P* value (overal)	*P* value (percreta vs increta)	*P* value (percreta vs accreta)	*P* value (increta vs accreta)
CXCL12	11.40 [6,12]	7.33 [5,12]	6.00 [4,12]	.008	.087	.007	.738
CXCR4	11.40 [6,12]	7.80 [6,12]	6.00 [3,12]	.002	.047	.001	.442
CXCR7	11.11 [5,12]	7.50 [5,12]	6.25 [3,12]	.003	.023	.005	1

Note

The values in brackets indicated the minimum and maximum values of the immunohistochemical scores, respectively.

### 3.3 The expression of plasma CXCL12, CXCR4 and CXCR7 in PAS and normal pregnant women

**Figure 2:**
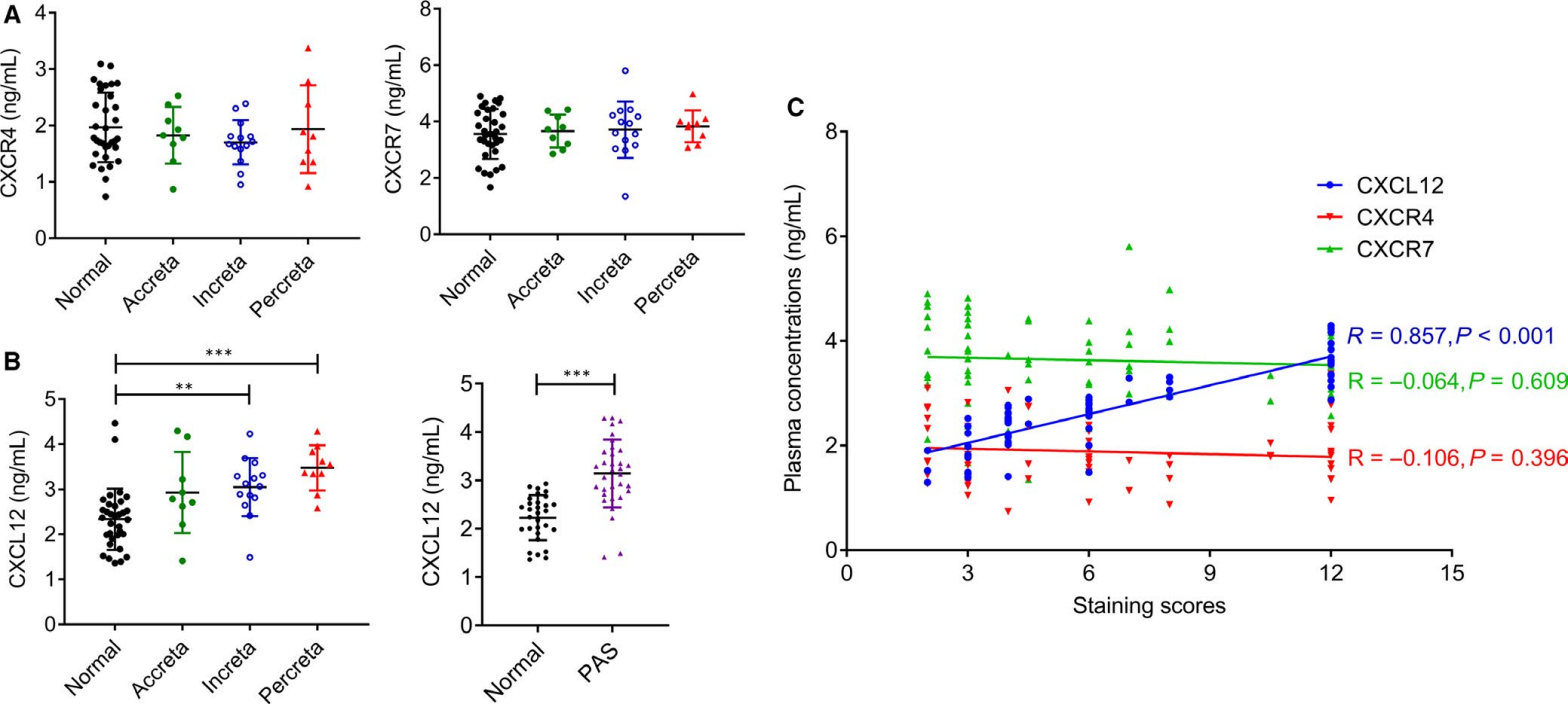
The expression of plasma CXCL12, CXCR4 and CXCR7 in PAS and normal pregnant women. (A) Plasma CXCR4 and CXCR7 levels in women with PAS and normal controls. (B) Plasma CXCL12 levels in women with PAS by depth of invasion. (C) Correlations between the immunohistochemical staining scores and plasma concentrations of CXCL12, CXCR4 and CXCR7 in women with PAS. *r* = Pearson's correlation coefficient. (**P ＜ .01, ***P ＜ .001)

The plasma concentrations of CXCL12, CXCR4 and CXCR7 were in accordance with normal distribution (Figure 2A‐B). Analysis result revealed that the plasma CXCL12 level in the PAS group was significantly increased (3.144 ± 0.701 ng/mL) than that in normal control group (2.207 ± 0.442 ng/mL) (*P* < .001) (Figure 2B, Table 5). We further divided the PAS into the placenta accreta, placenta increta and placenta percreta groups. We found that the level of plasma CXCL12 placenta percreta group (3.476 ± 0.500 ng/mL) was increased compared with that in the placenta increta group (3.047 ± 0.643 ng/mL) and placenta accreta group (2.927 ± 0.899 ng/mL), but there was no significant difference (*P* = .188) (Figure 2B, Table 6). Also, there was no statistical difference in plasma CXCR4/CXCR7 between PAS and normal pregnant women (CXCR4: *P* = .191; CXCR7: *P* = .484). In addition, the plasma CXCR4 and CXCR7 levels in placenta percreta group, placenta increta group, and the placenta accreta group showed no significant difference (CXCR4: *P* = .605; CXCR7: *P* = .807) (Figure 2A).

**Table 5:** Plasma CXCL12, CXCR4 and CXCR7 levels (ng/mL) in women with PAS and normal controls

	Case (n = 33)	Control (n = 33)	*t*	*P* value
CXCL12	3.144 ± 0.701	2.207 ± 0.442	5.942	<.001
CXCR4	1.805 ± 0.535	1.968 ± 0.617	1.337	.191
CXCR7	3.700 ± 0.784	3.568 ± 0.885	0.709	.484

**Table 6:** Plasma CXCL12, CXCR4 and CXCR7 levels (ng/mL) in women with PAS by depth of invasion

	Increta (n = 9)	Accreta (n = 14)	Percreta (n = 10)	*F*	*P* value
CXCL12	2.927 ± 0.899	3.047 ± 0.643	3.476 ± 0.500	1.767	.188
CXCR4	1.827 ± 0.503	1.703 ± 0.392	1.929 ± 0.734	0.512	.605
CXCR7	3.502 ± 0.967	3.536 ± 0.829	3.726 ± 0.670	0.216	.807

We analysed the relationship between CXCL12 expression in blood circulation and CXCL12 in placental trophoblast cells. The results present that CXCL12 expression in blood circulation of pregnant women with PAS was positively correlated with its expression in placental trophoblast cells (*r* = .857, *P* < .001) (Figure 2C). However, the levels of CXCR4 and CXCR7 in blood circulation of pregnant women with PAS were not significantly correlated with the those expressions in placental trophic cells (CXCR4: *r* = −.106, *P* = .396; CXCR7: *r* = −.064, *P* = .609) (Figure 2C).

### 3.4 Establishment of human trophoblastic HTR‐8/SVneo cell line with inhibition or overexpression of CXCL12, CXCR4 and CXCR7 proteins

The transfected HTR‐8/SVneo cells were observed under inverted microscope, the CXCL12 overexpression or interference plasmid was inserted green fluorescence reporter, and CXCR4 and CXCR7 overexpression or interference plasmid was added red fluorescence reporter (Figure S3). At the same time, HTR‐8/SVneo cells transfected with blank or scramble plasmid were used as negative controls (Figure S3).

**Figure 3:**
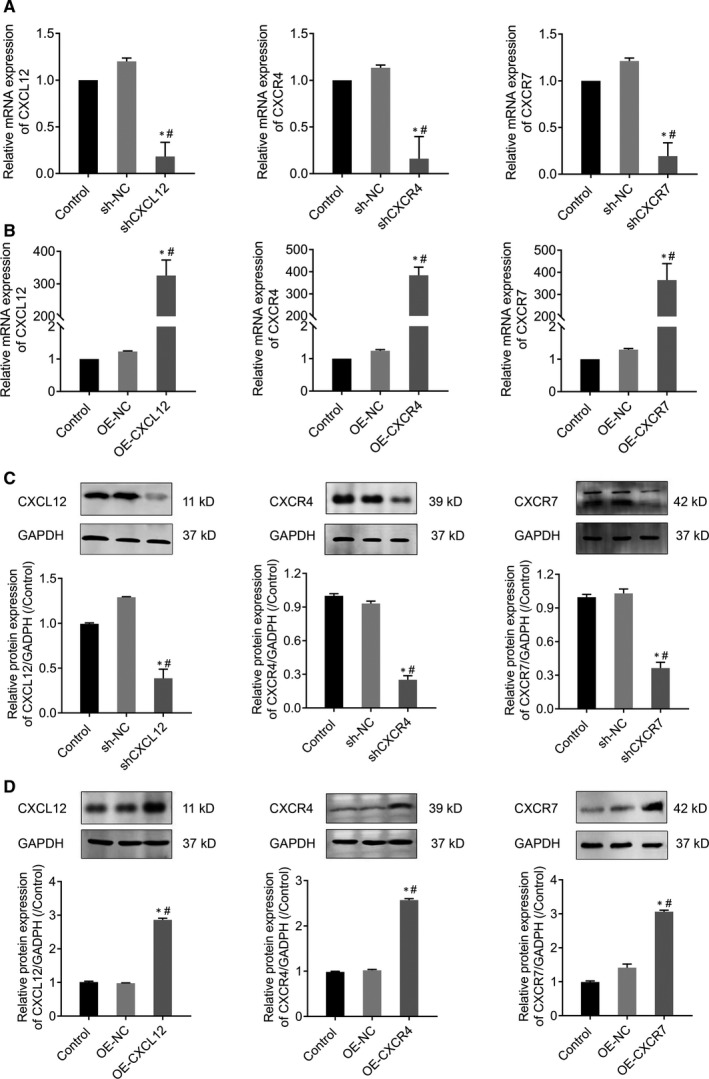
RT‐qPCR and Western blot detected CXCL12, CXCR4 and CXCR7 expression levels in CXCL12, CXCR4 and CXCR7 silenced or overexpression HTR‐8/SVneo cells. (A) RT‐qPCR detect transcriptional levels of silenced CXCL12, CXCR4 and CXCR7 in HTR‐8/SVneo (*P ＜ .05: Silenced cells vs Control; #*P* ＜ .05: Silenced cells vs sh‐NC). (B) RT‐qPCR detect transcriptional levels of overexpression CXCL12, CXCR4 and CXCR7 in HTR‐8/SVneo (*P ＜ .05: Overexpressed cells vs control; #*P* ＜ .05: Overexpressed cells vs OE‐NC). (C) Western blot to verify protein levels of silenced CXCL12, CXCR4 and CXCR7 in HTR‐8/SVneo (*P ＜ .05: Silenced cells vs Control; #*P* ＜ .05: Silenced cells vs sh‐NC). (D) Western blot to verify protein levels of overexpression CXCL12, CXCR4 and CXCR7 in HTR‐8/SVneo (*P ＜ .05: Overexpressed cells vs control; #*P* ＜ .05: Overexpressed cells vs OE‐NC)

To detect the efficiency of transfection, RT‐qPCR was used to verify the expression of CXCL12, CXCR4 and CXCR7 mRNA in each cell line after interference or overexpression. Results showed that the levels of CXCL12, CXCR4 and CXCR7 were significantly decreased in cells treated with shCXCL12, shCXCR4 and shCXCR7, respectively (*P* < .05) (Figure 3A). And the expressions of CXCL12, CXCR4 and CXCR7 in were significantly increased after overexpression with OE‐CXCL12, OE‐CXCR4 and OE‐CXCR7, respectively (*P* < .05) (Figure 3B).

Western blot further implicated that the levels of CXCL12, CXCR4 and CXCR7 proteins were significantly decreased in silence group of shCXCL12, shCXCR4 and shCXCR7 (*P* < .05) (Figure 3C), and significantly increased in OE‐CXCL12, OE‐CXCR4 and OE‐CXCR7 cells (*P* < .05) (Figure 3D).

### 3.5 CXCL12, CXCR4 and CXCR7 genes promote cell proliferation of HTR‐8/SVneo

**Figure 4:**
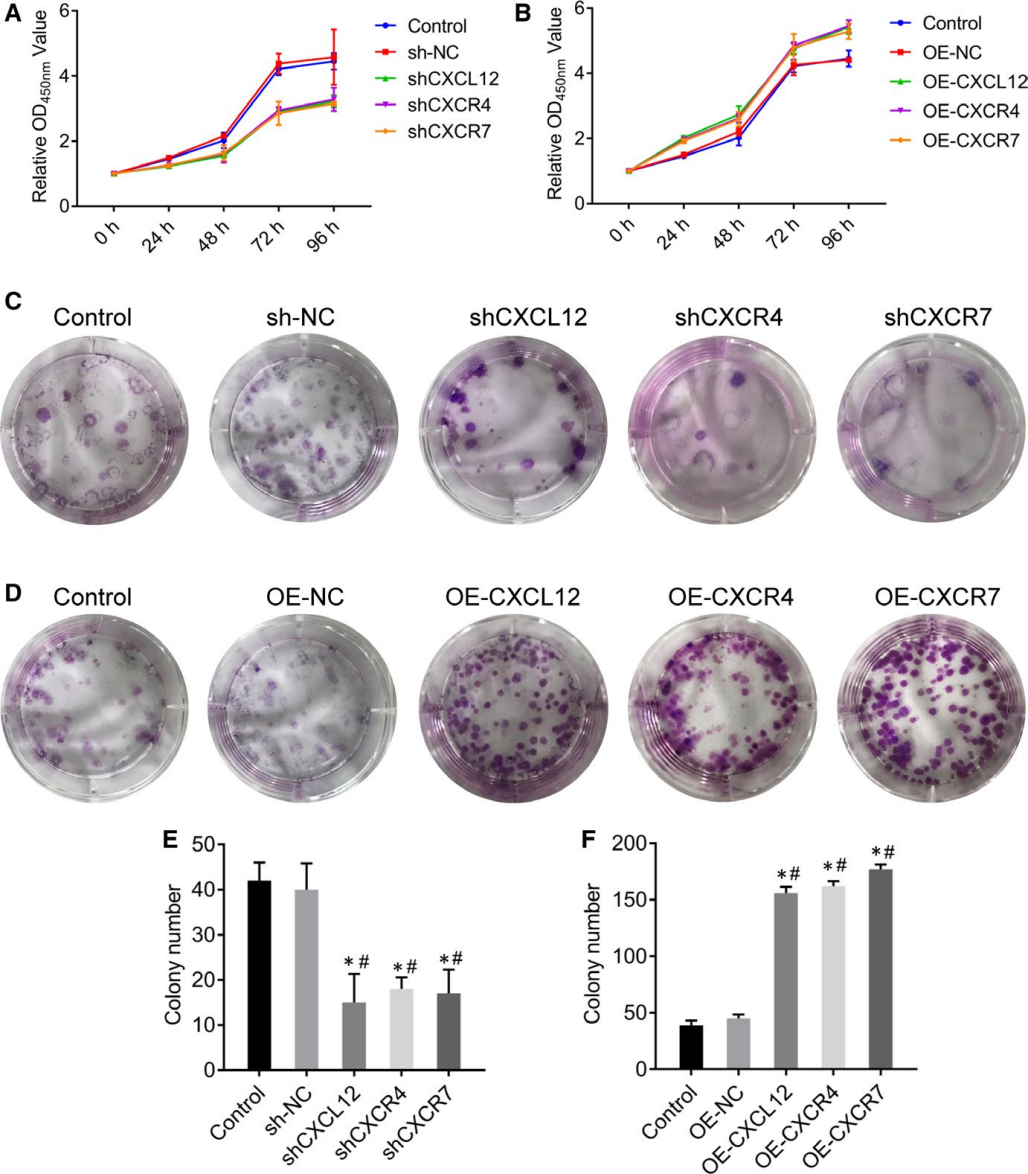
CXCL12, CXCR4 and CXCR7 genes promote cell proliferation of HTR‐8/SVneo. (A‐B) The effect of CXCL12, CXCR4 and CXCR7 silenced (A) or overexpression (B) in HTR‐8/SVneo on cell proliferation by CCK8 assays. (C‐F) The effect of CXCL12, CXCR4 and CXCR7 silenced (C, E) or overexpression (D, F) in HTR‐8/SVneo on cell proliferation by cloning formation experiment

To explore the function of CXCL12, CXCR4 and CXCR7 in cell proliferation, CCK‐8 assay was conducted. CCK‐8 results suggested that the cell proliferation rates of HTR‐8/SVneo were significantly decreased after the expressions of CXCL12, CXCR4 or CXCR7 genes were silenced (*P* < .05) (Figure 4A), indicating the down regulation of CXCL12, CXCR4 or CXCR7 inhibited the proliferation ability of HTR‐8/SVneo cells. By contrast, the proliferation rates were significantly increased in OE‐CXCL12, OE‐CXCR4 and OE‐CXCR7 groups (*P* < .05) (Figure 4B), which suggesting that overexpression of CXCL12, CXCR4 and CXCR7 genes enhanced cell proliferation of HTR‐8/SVneo.

Moreover, we performed cloning formation experiment to confirm this result. Results indicated that the cell proliferation rates of HTR‐8/SVneo were significantly decreased after the suppression of CXCL12, CXCR4 or CXCR7 (*P* < .05) (Figure 4C, E), but were significantly increased in OE‐CXCL12, OE‐CXCR4 and OE‐CXCR7 groups (*P* < .05) (Figure 4D, F). These results further demonstrated the involvement of CXCL12, CXCR4 and CXCR7 in cell proliferation of HTR‐8/SVneo.

### 3.6 CXCL12, CXCR4 and CXCR7 genes promote cell migration and invasion of HTR‐8/SVneo

**Figure 5:**
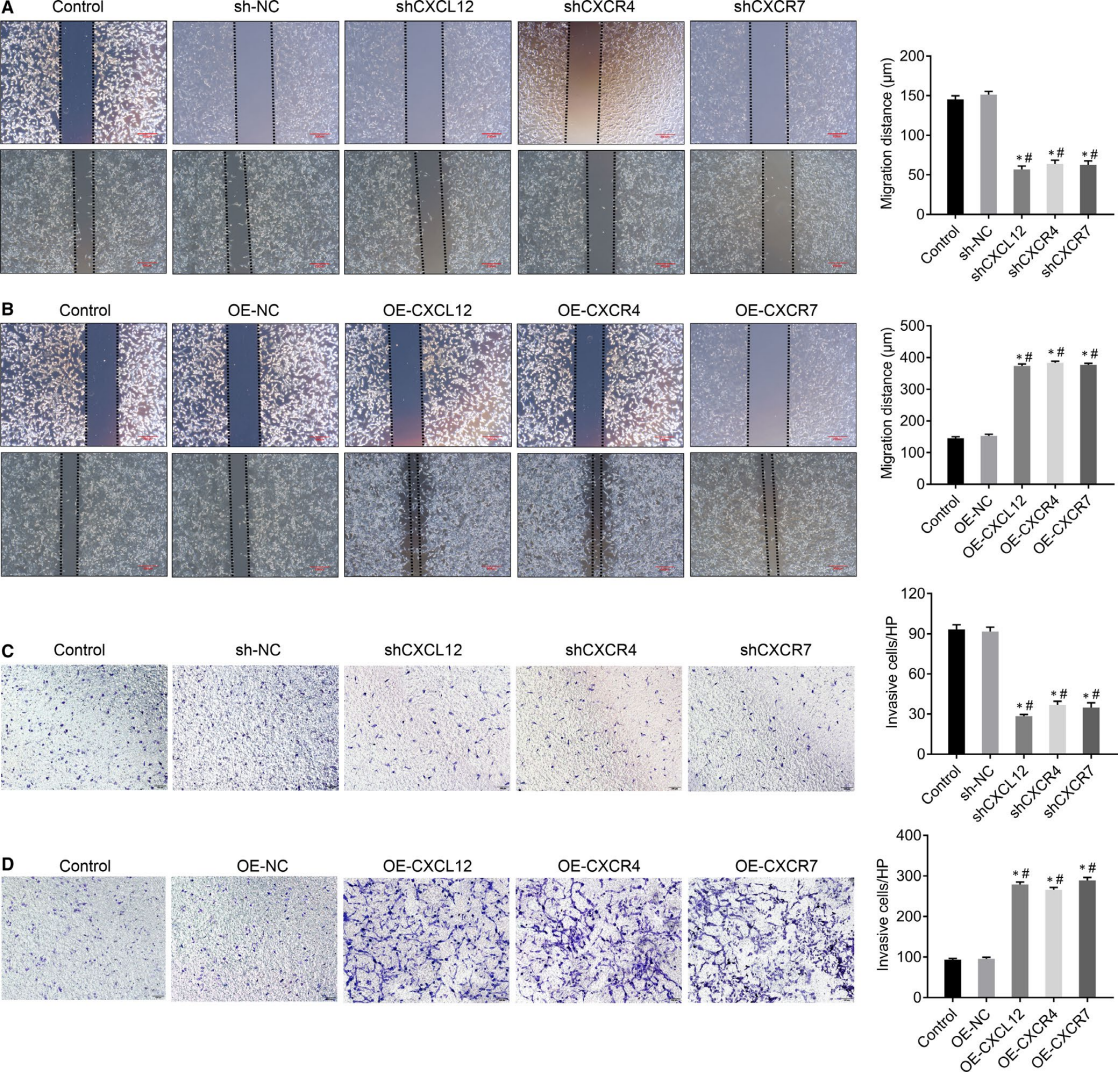
CXCL12, CXCR4 and CXCR7 genes promote cell migration and invasion of HTR‐8/SVneo. (A) The effect of silenced CXCL12, CXCR4 and CXCR7 in HTR‐8/SVneo on cell migration by wound healing assays (*P ＜ .05: Silenced cells vs Control; #*P *＜ .05: Silenced cells vs sh‐NC), Bar = 200 μm. (B) The effect of overexpression CXCL12, CXCR4 and CXCR7 in HTR‐8/SVneo on cell migration by wound healing assays (*P ＜ .05: Overexpressed cells vs control; #*P* ＜ .05: Overexpressed cells vs OE‐NC), Bar = 200 μm. (C) The effect of silenced CXCL12, CXCR4 and CXCR7 in HTR‐8/SVneo on cell invasion by transwell invasion assays. (*P ＜ .05: Silenced cells vs Control; #*P *＜ .05: Silenced cells vs sh‐NC), Magnification × 100. (D) The effect of overexpression CXCL12, CXCR4 and CXCR7 in HTR‐8/SVneo on cell invasion by transwell invasion assays. (*P ＜ .05: Overexpressed cells vs control; #*P* ＜ .05: Overexpressed cells vs OE‐NC), Magnification × 100

We evaluated the function of CXCL12, CXCR4 and CXCR7 in cell migration and invasion and carried out cell scratch assay and transwell assay. The cell scratch assay suggested that the cell migration distance of HTR‐8/SVneo cells was significantly reduced in silencing group (*P* < .05) (Figure 5A). Moreover, the cell migration ability of HTR‐8/SVneo cells was enhanced after overexpression of CXCL12, CXCR4 and CXCR7 genes (*P* < .05) (Figure 5B). Similarly, the transwell assay showed that invasion ability of HTR‐8/SVneo cells was significantly reduced in silenced group (*P* < .05) (Figure 5C), but significantly increased in overexpression group (*P* < .05) (Figure 5D). The above data suggested that CXCL12, CXCR4 and CXCR7 genes promote cell migration and invasion of HTR‐8/SVneo.

### 3.7 CXCL12, CXCR4 and CXCR7 have no obvious effect on cell apoptosis of HTR‐8/SVneo

**Figure 6:**
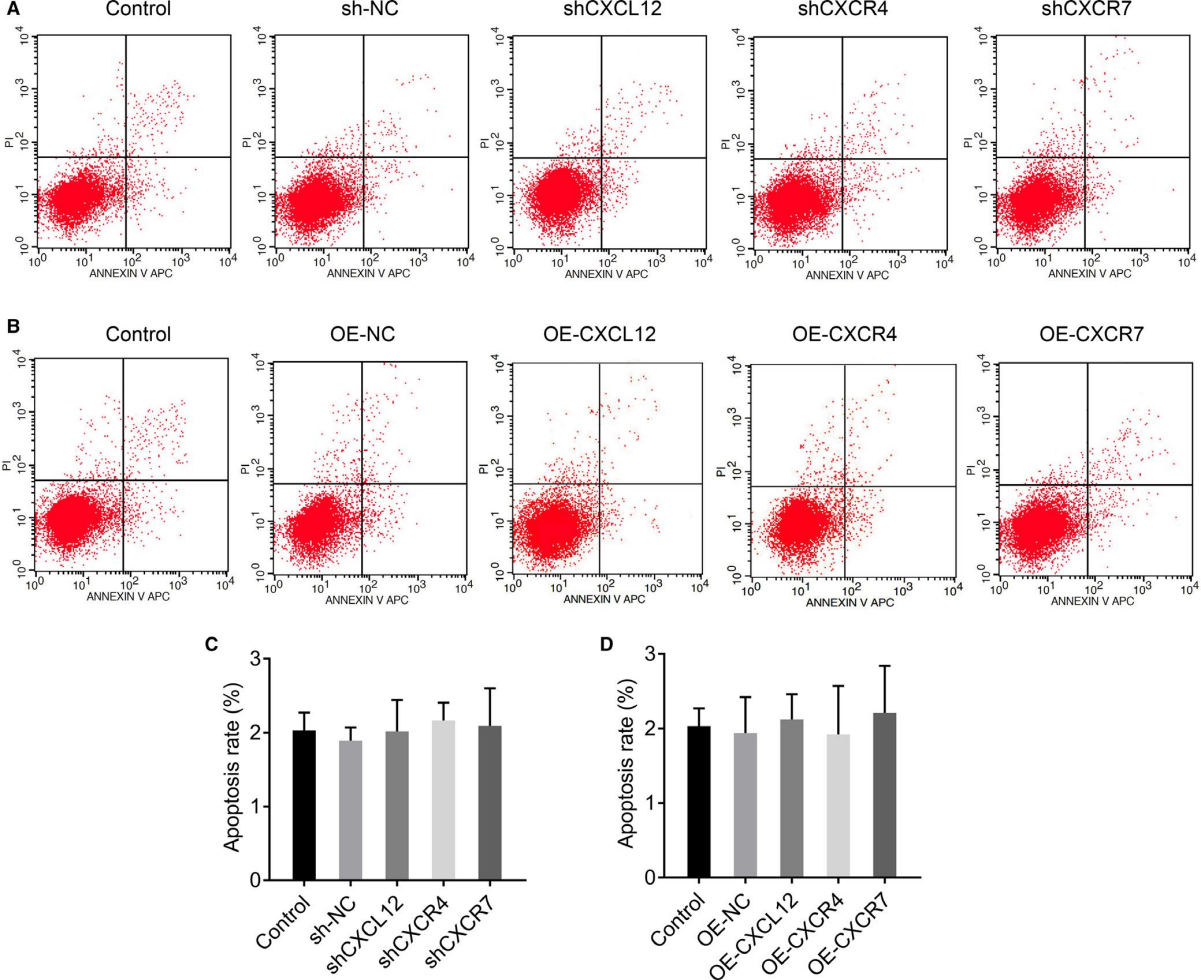
CXCL12, CXCR4 and CXCR7 have no obvious effect on cell apoptosis of HTR‐8/SVneo. (A, C) The effect of silenced CXCL12, CXCR4 and CXCR7 in HTR‐8/SVneo on cell apoptosis by flow cytometric assays (*P ＜ .05: Silenced cells vs Control; #*P *＜ .05: Silenced cells vs sh‐NC). (B, D) The effect of overexpression CXCL12, CXCR4 and CXCR7 in HTR‐8/SVneo on cell apoptosis by flow cytometric assays (*P ＜ .05: Overexpressed cells vs control; #*P* ＜ .05: Overexpressed cells vs OE‐NC)

Flow cytometry showed no significant difference of percentage of apoptosis between silencing groups (*P* > .05, Figure 6A, C) or overexpression groups (*P* > .05, Figure 6B, D), which demonstrated that CXCL12, CXCR4 and CXCR7 genes have no obvious effect on cell apoptosis of HTR‐8/SVneo.

### 3.8 CXCL12, CXCR4 and CXCR7 regulate cell invasion via in Rho/rock, PI3K/AKT signalling pathways

To further investigate the functional mechanism of CXCL12, CXCR4 and CXCR7, we analysed the downstream pathways or key proteins involved in cell invasion. Previous studies have reported that Rho GTP enzyme family regulated cytoskeletal changes and cell motility, including tumour cell migration and invasion, we then accordingly tested the levels of Rho GTPase family‐related protein RhoA, Rac1 and Cdc42, and key proteins involved in Rho/Rock and PI3K/AKT downstream signal pathway, including Rock, AKT, phosphorylated AKT (p‐AKT), myosin light chain (MLC) and phosphorylated myosin light chain (p‐MLC) after the overexpression or inhibition of CXCL12, CXCR4 and CXCR7 genes.

**Figure 7:**
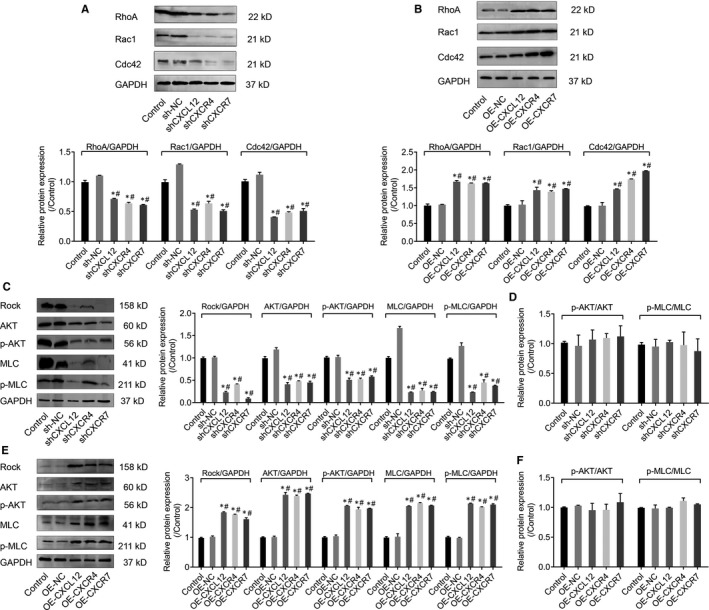
CXCL12, CXCR4 and CXCR7 regulates cell invasion via in Rho/rock, PI3K/AKT signaling pathways. (A) Western blot assays detected RhoA, Rac1 and Cdc42 protein expressions in CXCL12, CXCR4 and CXCR7 silenced HTR‐8/SVneo cells. The intensity of Western blot bands was quantified by image J software (*P ＜ .05: Silenced cells vs Control; #*P *＜ .05: Silenced cells vs sh‐NC). (B) Western blot assays detected RhoA, Rac1 and Cdc42 protein expressions in CXCL12, CXCR4 and CXCR7 overexpression HTR‐8/SVneo cells. The intensity of Western blot bands were quantified by image J software (*P ＜ .05: Overexpressed cells vs control; #*P* ＜ .05: Overexpressed cells vs OE‐NC). (C) The effect of silenced CXCL12, CXCR4 and CXCR7 on Rho/Rock and PI3K/AKT signaling pathways by Western blotting. (*P ＜ .05: Silenced cells vs Control; #*P* ＜ .05: Silenced cells vs sh‐NC), (D) The effect of silenced CXCL12, CXCR4 and CXCR7 on p‐Akt/Akt and p‐MLC/MLC ratios. (E) The effect of CXCL12, CXCR4 and CXCR7 overexpression on Rho/Rock and PI3K/AKT signaling pathways by Western blotting. (*P ＜ .05: Overexpressed cells vs Control; #*P *＜ .05: Overexpressed cells vs sh‐NC), (F) The effect of CXCL12, CXCR4 and CXCR7 overexpression on p‐Akt/Akt and p‐MLC/MLC ratios

Western blot results showed that the protein expression of RhoA, Rac1 and Cdc42 was significantly inhibited by transfection of shCXCL12, shCXCR4 and shCXCR7 (*P* < .05, Figure 7A). Meanwhile, the protein expression of Rock, AKT, p‐AKT, MLC and p‐MLC were also was significantly decreased (*P* < .05, Figure 7C). However, the protein expressions of RhoA, Rac1, Cdc42, Rock, AKT, p‐AKT, MLC and p‐MLC were all significantly increased in OE‐CXCL12, OE‐CXCR4 and OE‐CXCR7 groups (*P* < .05, Figure 7B, E). No significant change of ratios of p‐AKT/AKT and p‐MLC/MLC was found among different groups (*P* > .05, Figure 7D, F).

**Figure 8:**
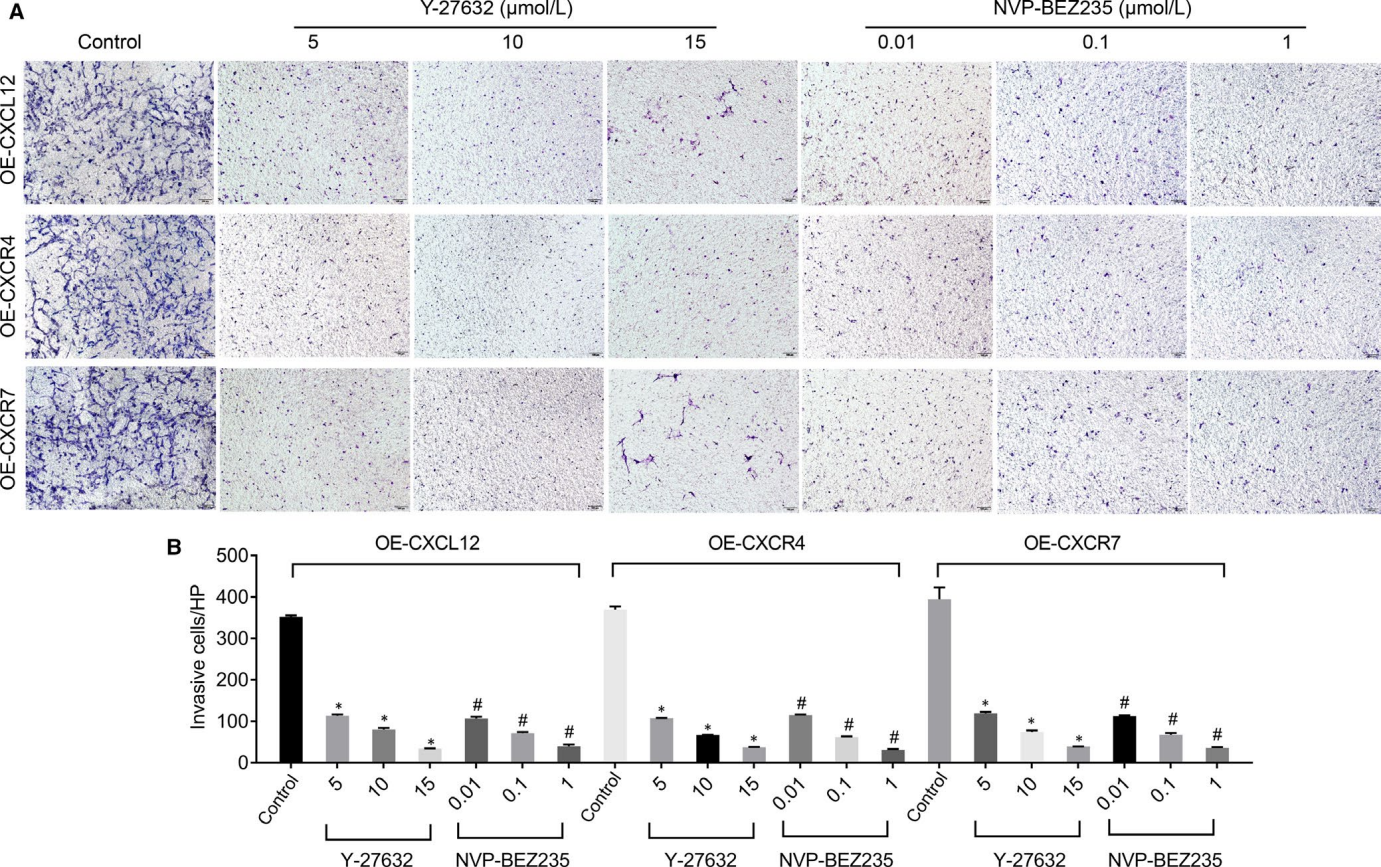
The effect of blocking Rho/Rock or PI3K/AKT pathways on the invasion of CXCL12, CXCR4 or CXCR7 stably overexpressed HTR‐8/SVneo cells by transwell invasion assay (A) and statistics (B). NVP‐BEZ235: PI3K inhibitor (#*P *＜ .05: vs Control), Y‐27632: Rock inhibitor (*P ＜ .05: vs Control)

To further validate the potential mechanism of CXCL12, CXCR4 and CXCR7 on cell invasion though Rho/rock, PI3K/AKT signalling pathway, we used Y‐27632 (Rock inhibitor) and NVP‐BEZ235 (PI3K inhibitor) to block signalling pathways, respectively and detected the cell invasive ability by transwell invasion experiments. Results suggested that after Rock or PI3K was inactivated, the cell invasive ability of wild‐type and CXCL12, CXCR4 and CXCR7 overexpressing groups was significantly impaired (*P* < .05, Figure 8A, B) Taken together, these results corporately indicated that CXCL12, CXCR4 and CXCR7 regulated the HTR‐8/SVNEO cell invasion through Rho/rock, PI3K/AKT signalling pathway.

## 4 DISCUSSION

The chemokine CXCL12 and the receptor CXCR4/CXCR7 are highly expressed in trophoblast cells due to PAS, and the expression intensity of CXCL12, CXCR4 and CXCR7 proteins is closely related to the invasion depth of trophoblast cells, which indicates that CXCL12‐CXCR4/CXCR7 may play an important role in the development of PAS. Previous studies have reported that the change of oxygen concentrations affected the proliferation, differentiation and invasion of trophoblast cells, while hypoxic conditions could promote the secretion of CXCL12, and subsequently stimulate the migration and invasion of trophoblast cells.^14^ Therefore, some researchers proposed that PAS was prone to occur when the placenta was attached to the lower part or the scar of the uterus, possibly due to local hypoxic concentration.^15^, ^16^ In this study, we found that CXCL12‐CXCR4/CXCR7 were closely associated with the excessive invasion of PAS trophoblast cells.

Recently, many studies have been actively identifying specific biochemical markers of placenta accreta. The early diagnosis of PAS in pregnant women can minimize the complications risk. It has proved that blood β‐HCG mRNA concentration was higher in PAS group than that placenta previa group,^17^ and several studies suggested that the human placental lactogen (hPL) mRNA was completely derived from the placenta trophoblasts and can predict intrapartal bleeding.^18^, ^19^ But these studies did not further analyse the exact invasion of depth.^20^ We found that the pathological diagnosis of PAS maternal blood CXCL12 levels was significantly increased based on the data from gestational age paired case‐control study, and blood CXCL12 levels was positively correlated with placental trophoblast CXCL12 protein expression. Therefore, it demonstrated that the high expression of placental trophoblastic chemokine CXCL12 might be originated from blood CXCL12. This suggests that blood CXCL12 has the potential to become a predictive biochemical markers of PAS, and our data indicate the possibility of blood CXCL12 for distinguishing invasive depth of trophoblast cells.

The CXCl12‐CXCR4/CXCR7 axis plays an important role in many angiogenesis related diseases, and it has been reported that changes in chemokines or their receptors can be detected in circulating blood. Studies have also reported elevated CXCL12 levels in the placenta and circulating blood of women with preeclampsia.^21^ Kim et al dynamically monitored the serum CXCL12 levels of 100 patients undergoing cardiopulmonary bypass before surgery, during myocardial ischaemia, reperfusion and after surgery, and found that CXCL12 level was significantly increased during myocardial ischaemia, but had no significant effect during cardiac reperfusion, which means that CXCL12 serum levels are inversely correlated with organ dysfunction.^22^ In addition, Nadimi et al revealed that serum levels of CXCL12 and CXCL10 were significantly correlated with the severity of coronary artery occlusion.^23^ Matsuoka et al collected peripheral blood of 206 patients before angiography and detected CXCL12 level in plasma, and present that CXCL12 level in patients with ischaemic stroke was higher than that in patients without stroke.^24^ In this study, we found that CXCL12 level in the blood of pregnant women with PAS was significantly increased, suggesting a potential biochemical predictor of PAS. To sum up, the advantages of chemotactic factor CXCL12 not only have the potential to become the new biomarkers to predict PAS, but also participates in the biology of the placenta. It is still of great significance to dynamically monitor CXCL12 levels at different gestational stages and to further explore the pathogenesis of placenta accrete which provide guidance for appropriate treatment timely. The chemokine CXCL12, belonging to the chemokine CXC subfamily, was identified as pre‐B‐cell growth‐stimulating factor.^25^, ^26^ In recent years, the role of chemokine CXCL12 in mediating the regulation of maternal‐fetal interface or placental growth and development has attracted much attention.^11^ Previous study showed that CXCLl2 and CXCR4 were related to the regulation of trophoblast proliferation, and promotion of trophoblasts differentiation into invasive types, which participated in the early invasion of the placenta, as well as the growth and development of the embryo.^27^ The concentration of autocrine and paracrine CXCL12 and the receptor CXCR4 in trophoblast cells were changed under different oxygen concentration environments.^16^ And the trophoblast migration and invasion ability changed accordingly to different CXCL12 concentrations.^28^ Above results suggested that CXCLl2 can promote the migration of trophoblasts to the aponeurosis and blood vessels, the invasion of placenta, uterine‐placental vascular remodelling, immune tolerance and complete a series of pregnancy physiological processes,^29^, ^30^, ^31^ In this study, we found high expressions of CXCL12, CXCR4 and CXCR7 proteins in the trophoblasts at the abnormally invasive site and were associated with the invasive depth. Overexpression of CXCL12/CXCR4/CXCR7 enhanced cell proliferation, migration and invasion. On the contrary, inhibition of CXCL12/CXCR4/CXCR7 limited cell proliferation, migration and invasion, but not affected on cell apoptosis, which exerts regulation via other mechanisms. In conclusion, CXCL12‐CXCR4/CXCR7 are involved in the regulation of trophoblast proliferation, migration and invasion, and participate in PAS of trophoblast excessive invasion, which provide insights for the study of PAS mechanism.

The Rho GTP enzyme family proteins are classified into four major classes, RhoA, Racl, Cdc42 and GTPase‐deficient.^32^ Most Rho GTP enzymes have GTPase activity and are involved in changing cell morphology, regulating cell‐matrix accreta and cytoskeletal reorganization, thereby regulating cell migration and invasion.^33^ In our study, we found that the expressions of CXCL12, CXCR4 and CXCR7 are positively correlated with the expression of Rho GTPase family‐related proteins RhoA, Rac1 and Cdc42, suggesting that CXCL12, CXCR4 and CXCR7 in trophoblastic cells regulated cell invasion probably through the Rho GTPase family proteins. Rho‐associated coiled‐coil forming protein kinase (ROCK) is an important effector downstream of Rho.^34^ Rho and ROCK can increase the phosphorylation level of myosin light chain (MLC) and thereby enhance the contractile force of myosin‐myosin to promote the migration of target cells in ECM.^35^ Kent et al found that the CXCL12/CXCR4 axis up‐regulated the expression of MMPs by activating the P13K/AKT/FOSL1 signalling pathway, which enables the trophoblasts to acquire strong migration ability.^36^ In our study, we showed that the invasion of trophoblast cells was significantly inhibited by using Rock inhibitor (Y‐27632) and PI3K inhibitor (NVP‐BEZ235). These results coordinately suggest that CXCL12, CXCR4 and CXCR7 modulated of trophoblast invasion via Rho/Rock and PI3K/AKT signalling pathways, based on the evidence that the up‐regulation of CXCL12, CXCR4 and CXCR7 markedly elevated the protein expressions of RhoA, Rac1, Cdc42, Rock, AKT, p‐AKT, MLC and p‐MLC and vice versa (down‐regulation of CXCL12, CXCR4 and CXCR7 led to the decrease of protein expressions), along with no significant change of ratios of p‐AKT/AKT and p‐MLC/MLC, in order to determine changes in not only total but also activation level in the signalling pathway. The limitation in our study still exists that pathways due to the budget and ethic issue, while the samples from early and middle stages of gestation will be collected and tested in further research. Our current data reveal that, in PAS, CXCL12, CXCR4 and CXCR7 regulate cell invasion via in Rho/rock, PI3K/AKT signalling pathways, but the potential pathways regarding the pathogenesis remain to be identified. The limitation in our study still exists that only the expressions of membrane‐bound receptors in plasma were detected as a less invasiveness way on pregnant women, and the levels in the tissue as well as the potential therapeutic effect should be further investigated by using animal model.

**Figure 9:**
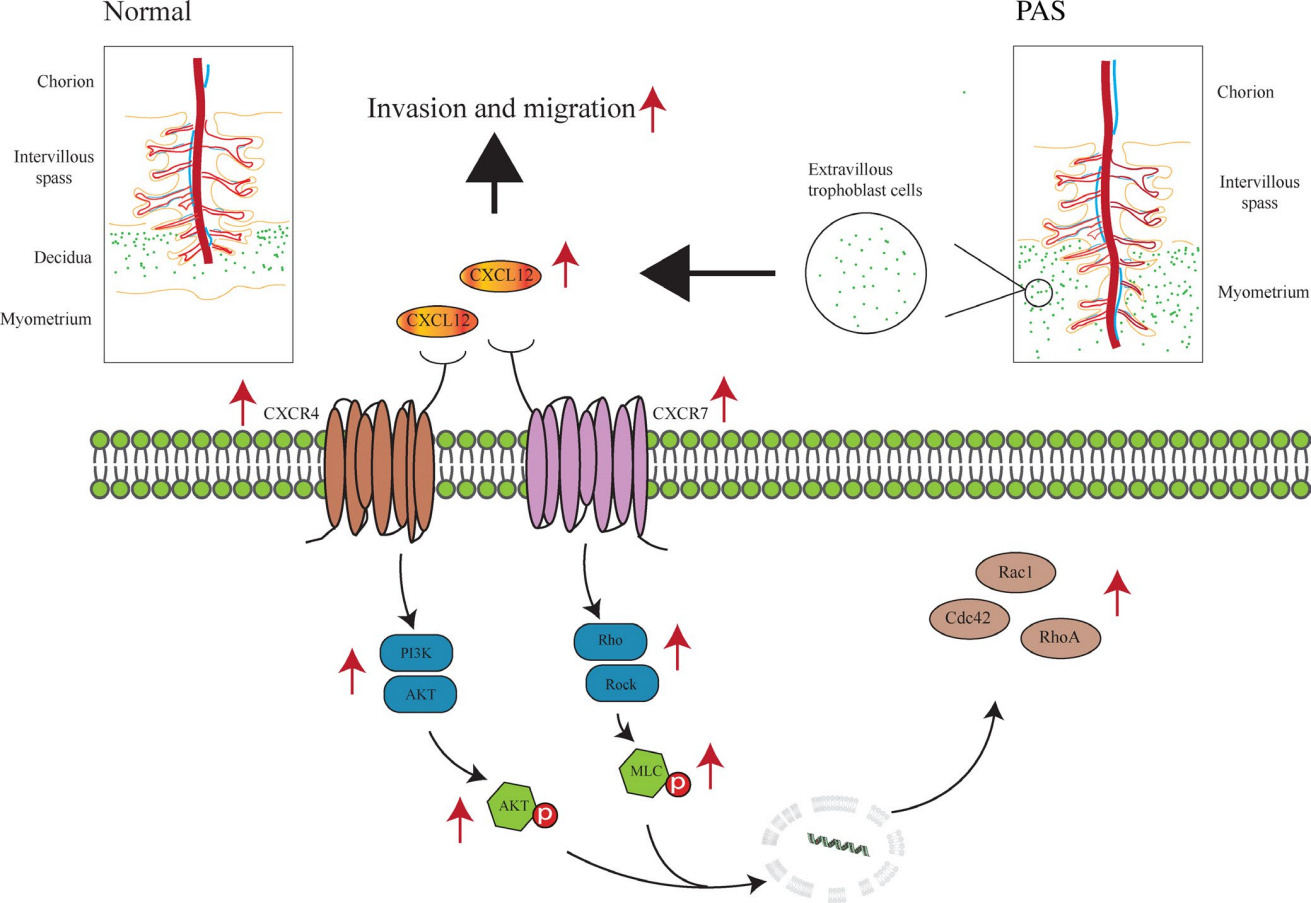
Schematic representation of CXCL12 and receptor CXCR4/CXCR7 in placenta accrete spectrum disorders (PAS). PAS increased expression of chemokine CXCL12 and receptor CXCR4/CXCR7 in extravillous trophoblastic cells. CXCL12, CXCR4 and CXCR7 could up‐regulate the expression of RhoA, Rac1 and Cdc42 proteins to promote the migration and invasion of extravillous trophoblastic cells and ultimately form PAS through increase of phospho‐MLC and phospho‐AKT in Rho/rock and PI3K/AKT signaling pathway

In conclusion, we demonstrate that CXCL12 and CXCR4/CXCR7 play an important role in altering cell morphology, cell‐matrix accreta and cytoskeletal reorganization. The expressions of CXCL12, CXCR4 and CXCR7 in extravillous trophoblastic cell are significantly increased and further give rise to the up‐regulation of CXCL12 in maternal blood. The chemokine and its receptor can activate the phosphorylation and elevate the expression of MLC and AKT proteins of the Rho/rock, PI3K/AKT signalling pathway. The up‐regulation of RhoA, Rac1 and Cdc42 proteins ultimately promotes the migration and invasion of extravillous trophoblastic cell and facilitates the formation of the PAS compare with the normal placenta (Figure 9). In this regard, the chemokine CXCL12 and its receptors CXCR4/CXCR7 become potential therapeutic targets. The block of the interaction of CXCL12 and its receptors CXCR4/CXCR7, or the deactivation of the downstream Rho/Rock and PI3K/AKT signalling pathway, might be a new approach to approach to the treatment of PAS.

## CONFLICT OF INTEREST

The authors declare that they have no conflict of interest.

## AUTHOR’S CONTRIBUTION

Yu Long, Yonghua Jiang and MuJun Li conceived the study, designed experiments and wrote the manuscript. Yu Long, Yonghua Jiang, Jingjing Zeng and Yiwu Dang provided new tools/reagents and performed experiments. Yue Chen, Jueying Lin, Hongwei Wei, Hongwei Xia, Junqing Long and Cuizhen Luo developed new software, collection of clinical specimens and data. Zhiwei Chen and Yaling Huang analysed data and made manuscript revisions. All authors read and approved the final manuscript.

## Data Availability

The data that support the findings of this study are available from the corresponding author upon reasonable request.
