# Supplementary figures and images for: The expression and biological function of chemokine CXCL12 and receptor CXCR4/CXCR7 in placenta accreta spectrum disorders
Source: J Cell Mol Med. 2020 Jan 28;24(5):3167–82. doi: 10.1111/jcmm.14990 (PMC7077540; doi:10.1111/jcmm.14990)

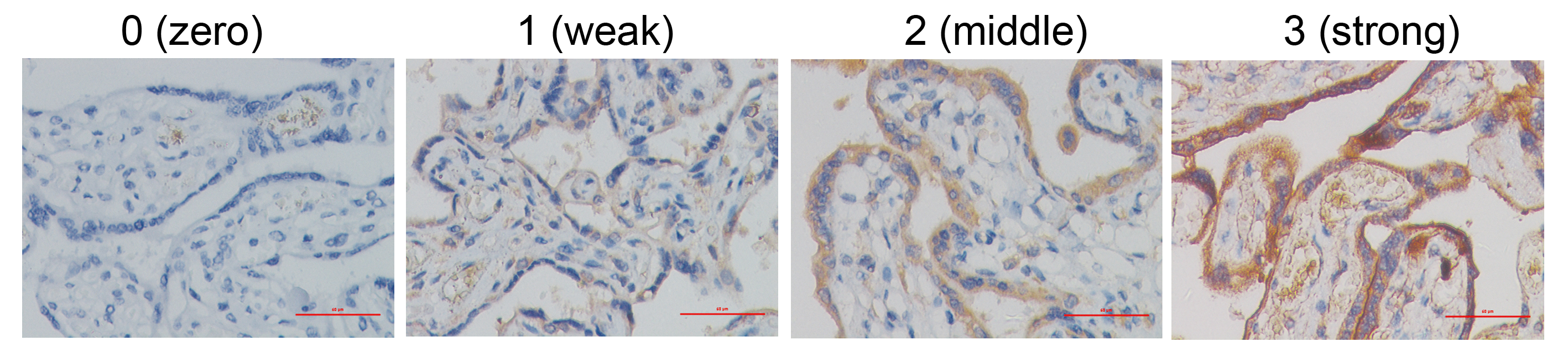

Supplement: Supplementary file 1 [file JCMM-24-3167-s001.tif]

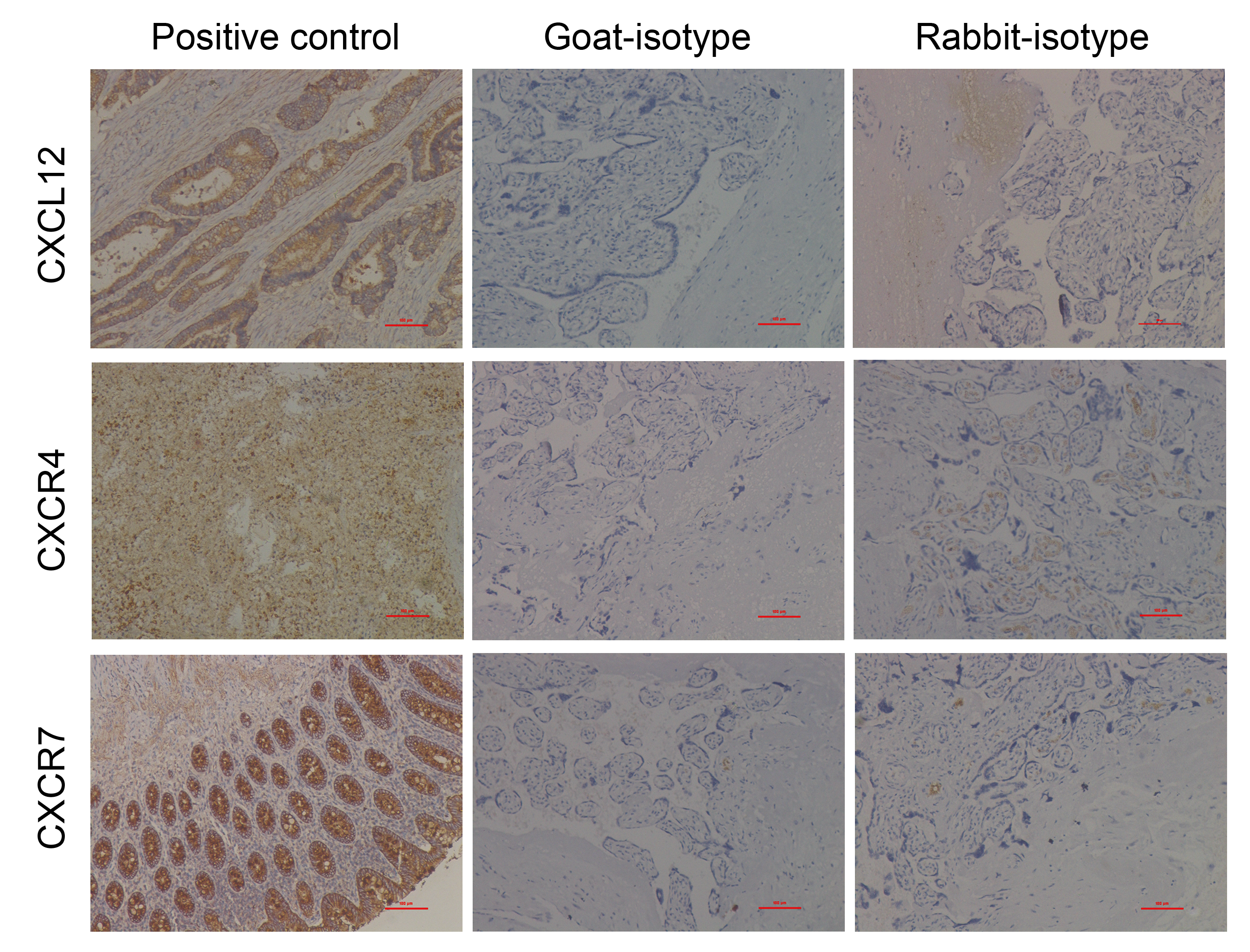

Supplement: Supplementary file 2 [file JCMM-24-3167-s002.tif]

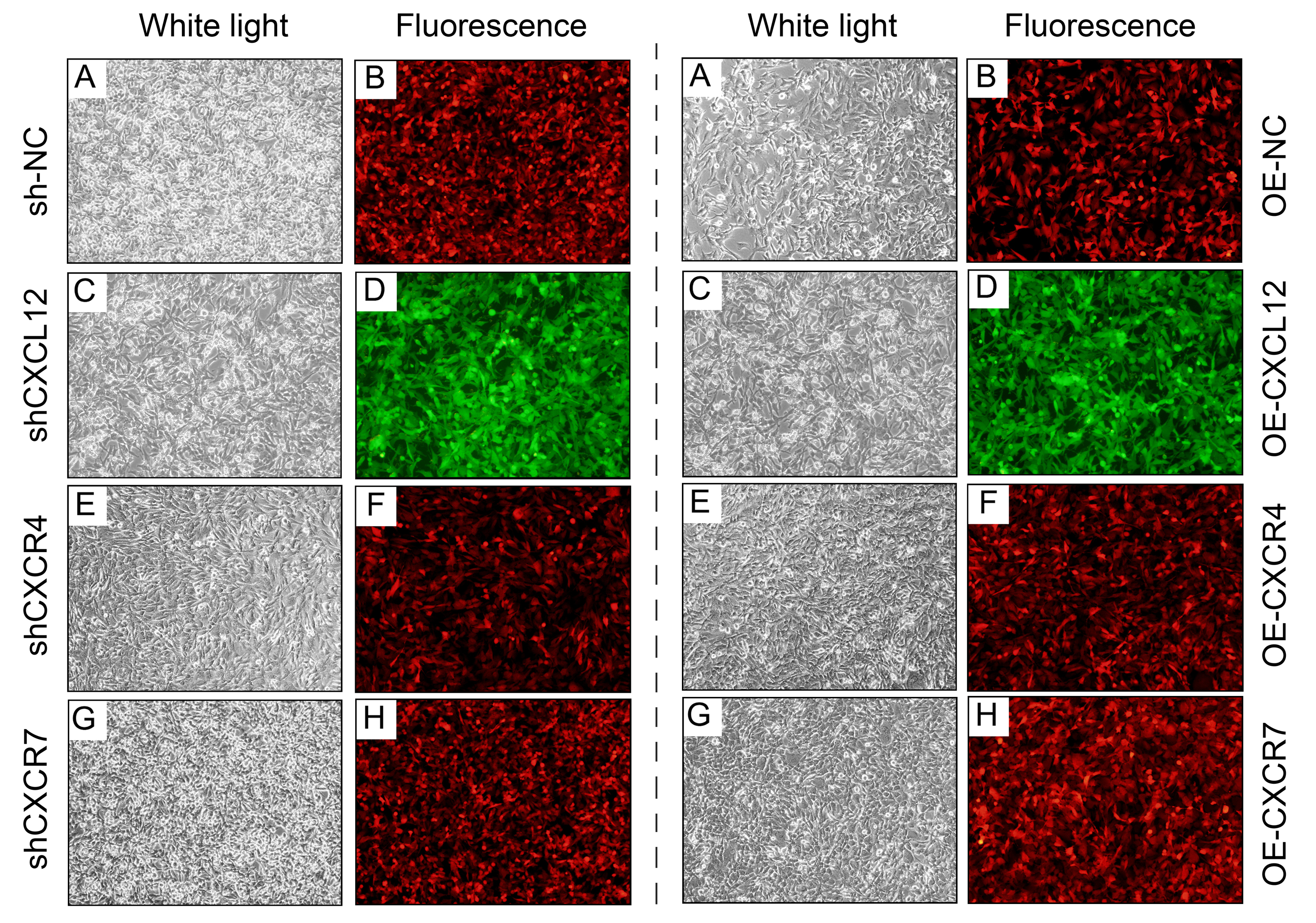

Supplement: Supplementary file 3 [file JCMM-24-3167-s003.tif]
